# Correction: Insects (*Thrips hawaiiensis* (Morgan)) change the stereochemical configuration of 1-phenylethanol emitted from tea (*Camellia sinensis*) flowers

**DOI:** 10.1039/d0ra90047h

**Published:** 2020-05-04

**Authors:** Ying Zhou, Lanting Zeng, Yinyin Liao, Fang Dong, Qiyuan Peng, Jianlong Li, Jinchi Tang, Naoharu Watanabe, Ziyin Yang

**Affiliations:** Guangdong Provincial Key Laboratory of Applied Botany, Key Laboratory of South China Agricultural Plant Molecular Analysis and Genetic Improvement, South China Botanical Garden, Chinese Academy of Sciences Xingke Road 723, Tianhe District Guangzhou 510650 China zyyang@scbg.ac.cn +86-20-38072989; University of Chinese Academy of Sciences No. 19A Yuquan Road Beijing 100049 China; Guangdong Food and Drug Vocational College Longdongbei Road 321, Tianhe District Guangzhou 510520 China; Tea Research Institute, Guangdong Academy of Agricultural Sciences, Guangdong Provincial Key Laboratory of Tea Plant Resources Innovation and Utilization Dafeng Road 6, Tianhe District Guangzhou 510640 China; Graduate School of Science and Technology, Shizuoka University 3-5-1 Johoku, Naka-ku Hamamatsu 432-8561 Japan

## Abstract

Correction for ‘Insects (*Thrips hawaiiensis* (Morgan)) change the stereochemical configuration of 1-phenylethanol emitted from tea (*Camellia sinensis*) flowers’ by Ying Zhou *et al.*, *RSC Adv.*, 2017, **7**, 32336–32343. DOI: 10.1039/C7RA03219F.

The authors regret that incorrect versions of Fig. 4 and 6 were included in the original article. The correct versions of Fig. 4 and 6 are presented below.

**Fig. 4:**
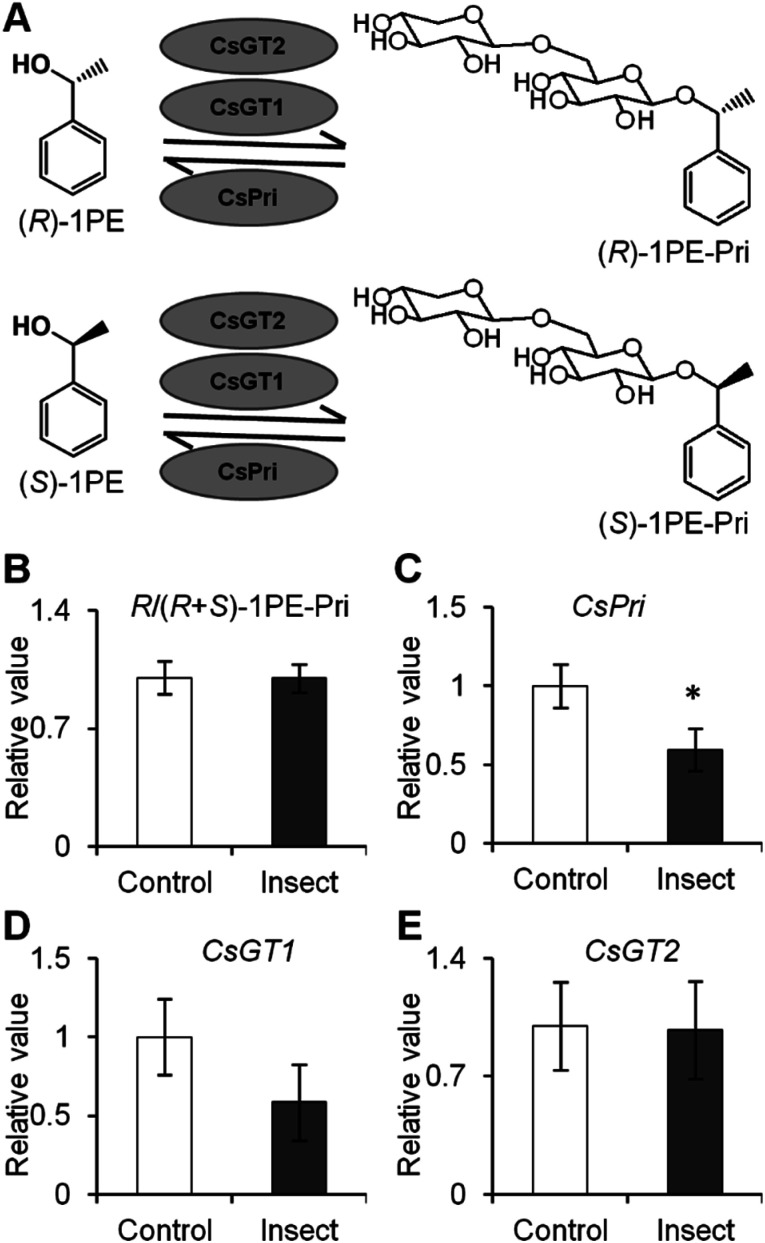
Effect of *Thrips hawaiiensis* (Morgan) attacks on (*R*)-/(*R* + *S*)-1PE-Pri ratio and expression levels of *CsGT1*, *CsGT2*, and *CsPri* in *C. sinensis* flowers. (A) Schemes of transformation between (*R*)-/(*S*)-1PE-Pri and (*R*)-/(*S*)-1PE. 1PE, 1-phenylethanol; 1PE-Pri, 1PE-β-primeveroside; GT, glycosyltransferases; Pri, β-primeverosidase. (B) Effect of *T. hawaiiensis* attacks on (*R*)-/(*R* + *S*)-1PE-Pri in *C. sinensis* flowers. Control, undamaged flowers. Insect, *T. hawaiiensis*-damaged flowers. The ratio of (*R*)-1PE-Pri to (*R* + *S*)-1PE-Pri in control was set as 1. (C–E) Effect of *T. hawaiiensis* attacks on expression levels of *CsPri*, *CsGT1*, and *CsGT2* in *C. sinensis* flowers. Transcript abundance was calculated based on the difference in cycle threshold (*C*_t_) values between target gene and internal reference gene transcripts by the normalized relative quantitation 2^−ΔΔ*C*_t_^ method. The expression level in control was set as 1. Significant differences between control and insect are indicated (*p ≤ 0.05). Data represent the mean value ± standard deviation of three independent experiments performed in triplicate.

**Fig. 6:**
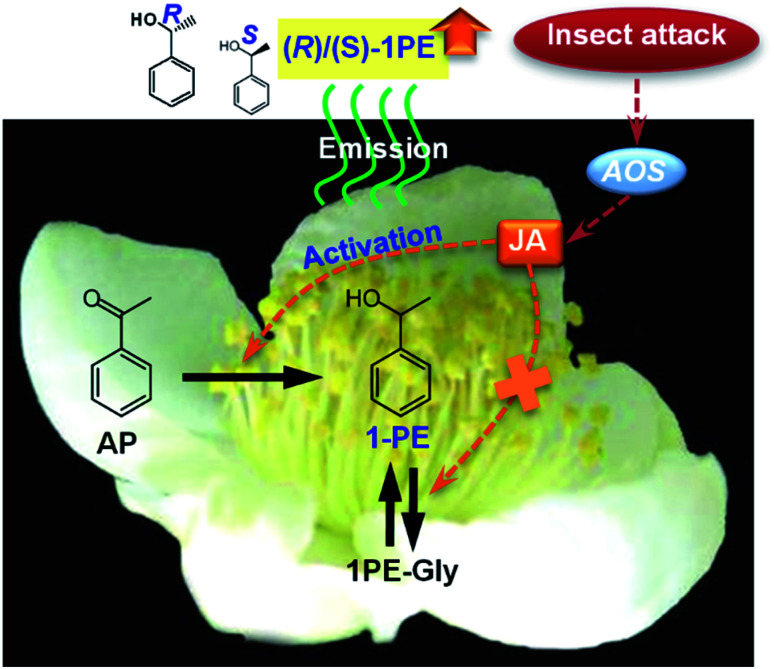
Proposed schematic model of change in ratio of (*R*)-1PE to (*S*)-1PE emitted from *C. sinensis* flowers exposed to insect attacks. AP, acetophenone; 1PE, 1-phenylethanol; 1PE-Gly, glycosides of 1-phenylethanol; JA, jasmonic acid; AOS, allene oxide synthase.

The Royal Society of Chemistry apologises for these errors and any consequent inconvenience to authors and readers.

## Supplementary Material

